# HYPERTENSION TREATMENT IN U.S. LONG-TERM NURSING HOME RESIDENTS WITH AND WITHOUT DEMENTIA

**DOI:** 10.1093/geroni/igz038.2946

**Published:** 2019-11-08

**Authors:** Kenneth Boockvar, Wei Song, Sei Lee, Orna Intrator

**Affiliations:** 1 Icahn School of Medicine at Mount Sinai, New York, New York, United States; 2 University of Rochester, Rochester, New York, United States; 3 University of California, San Francisco, San Francisco, California, United States

## Abstract

Background: Treatment of hypertension in individuals with dementia has benefit-harm tradeoffs. The objectives of this study were to describe patterns of antihypertensive treatment in U.S. nursing home (NH) residents and determine the association between intensity of antihypertensive treatment and outcomes important for persons with dementia. Methods: Medicare claims and Minimum Data Set (MDS) assessments were used to capture information on dementia, hypertension, comorbid conditions, cognitive function, activities of daily living (ADLs), and hospitalizations in Medicare-enrolled long-term care residents in 2013. Intensity of hypertension treatment was defined as number of antihypertensive drugs received on the date of an index MDS according to Medicare part D claims. Outcomes ascertained were hospitalizations, ADL decline, and mortality within 180 days of the index MDS. Results: A total of 262,285 residents had both hypertension and dementia. Residents were 85.7 years old on average and 77% female. At baseline, 30%, 40%, and 30% were receiving 0, 1, and 2+ antihypertensive medications, respectively. In multivariable logistic regression models, increased intensity of antihypertensive treatment was associated with increased hospitalization among residents with dementia (OR 1.03 per added medication; 95%CI 1.01-1.04), but decreased hospitalization among residents without dementia (OR .98 per added medication; 95%CI .97-1.00; p-value for interaction < .0001). In residents with and without dementia, increased intensity of antihypertensive treatment was associated with increased hospitalization for cardiovascular diseases, but small decreases in ADL decline and mortality. Conclusion: Study findings suggest that long-term NH residents with hypertension and dementia may not experience benefit from more intensive blood pressure control.

